# Understanding changes to perceived socioeconomic and psychosocial adversities during COVID-19 for UK freelance cultural workers

**DOI:** 10.1080/09548963.2022.2082270

**Published:** 2022-05-30

**Authors:** Katey Warran, Tom May, Daisy Fancourt, Alexandra Burton

**Affiliations:** Research Department of Behavioural Science and Health, Institute of Epidemiology & Health Care, University College London, London, UK

**Keywords:** Freelancers, self-employed, COVID-19, qualitative, mental health, precarity

## Abstract

There is a dearth of qualitative research exploring how freelancers working in the cultural industries have been affected during COVID-19. In particular, there is a lack of research exploring how socioeconomic and psychosocial adversities may have changed or evolved, and how these changes have been perceived and subjectively experienced by freelance cultural workers. This study builds on qualitative interviews carried out in July–November 2020 (*n* = 20) by exploring findings from follow-up interviews conducted in May–July 2021 (*n* = 16). It presents an inequality of experiences connected to a range of socioeconomic and psychosocial factors, showing how some freelancers experienced small changes (e.g. to the kind of work carried out), with others experiencing major changes (e.g. leaving the sector completely). It concludes with a call for highly bespoke financial and psychological support, as well as a need to rethink what cultural value is for this workforce in the “new normal”, considering changing valuing processes.

## Introduction

In response to the coronavirus-19 (COVID-19) pandemic, the cultural industries saw rapid and major changes in relation to how culture is produced and consumed. The first UK lockdown in March 2020 prompted the mass closure of arts and cultural venues and cancellation of in-person events, with venues then having to adhere to changing mandated restrictions as the pandemic continued. Many venues had to operate at reduced capacity, adapt their offering, or cease to operate at all. These changes fluctuated in line with the easing and tightening of social distancing throughout the pandemic, with many cultural venues oscillating between in-person and digital offerings, alongside hybrid models. The impact of these ongoing changes provided many challenges for the sector, including fewer employment opportunities (Office for National Statistics, 2021), but also increased some opportunities in relation to digital production (Radermecker, 2020), and home-based arts engagement (Bu et al., 2021a).^1^

A freelance career in the cultural sector has long been recognised as a path that may involve substantial financial or psychological risk, including periods of low or unpaid work, with social, cultural, and symbolic capital also important to “making it” (Gerber, 2017; Gross & Musgrave, 2020; Menger, 2001). The conditions in which freelance cultural workers exist are precarious and typified by inequalities, as has been widely acknowledged within research in the last decade (Brook et al., 2018; Comunian & England, 2020; Morgan & Nelligan, 2017; Scharff, 2017). The flux of the pandemic has exacerbated many of these pre-existing challenges, particularly for those whose careers place in-person work at the heart of their offering, such as those that rely on live performances (Comunian & England, 2020). Research conducted in 2020 showed that the first UK lockdown adversely affected employment and income for this group, with fewer opportunities available and income loss common (Creative Scotland, 2020; Cultivator, 2020; Oxford Economics, 2020; ScreenSkills, 2020). For example, survey results published in April 2020 exploring the impact of the pandemic on creative freelancers in Wales (*n* = 237) found that 85% of freelancers reported that their work either decreased sharply (25%) or dried up completely (60%) (Komorowski & Lewis, 2020). Different demographic groups may have experienced financial challenges during the pandemic differently too, with women and younger freelancers more adversely affected (Florisson et al., 2021). Further, employment precarity for freelancers during the first UK lockdown resulted not only in financial implications but negative consequences for mental health, social relationships and sense of identity (May et al., 2022). This supports research showing a perceived link between financial hardship and lower wellbeing for performing arts professionals (Spiro et al., 2021).

Ongoing quantitative research has explored these work changes across different stages of the pandemic, for example showing how the numbers of freelancers has steadily declined since the onset of COVID-19 (Florisson et al., 2021). However, the nuances behind these changing employment numbers in relation to what work opportunities have reduced and why freelancers may choose to leave the sector is less known, with very few studies exploring the changing psychosocial impact of the pandemic on freelancers. Further, the studies that do exist have primarily focused on the music industry (Cohen & Ginsborg, 2021; Daffern et al., 2021; Ptatscheck, 2021), with a lack of focus on other cultural industries. Understanding psychosocial impact for a range of cultural workers is important because the complex “social” aspects of a career in the cultural sector are viewed as essential for securing work opportunities and contributing to perceived work enjoyment whereby work colleagues are often considered friends and sources of social support (Gerber, 2017; Lingo & Tepper, 2013). Understanding how to support freelancers during the pandemic is underexplored, such as what strategies may be used to cope with work opportunity decline, changing social networks, and mental health impact. Given that major disruptions to work life can lead to serious mental health consequences (Marmot, 2005; Marmot et al., 2012), understanding the impact of ongoing disruptions is worthy of further attention.

Thus, given the rapid societal change taking place throughout COVID-19, this research aimed to explore how perceived socioeconomic and psychosocial experiences may have evolved or changed over the course of the pandemic for freelance cultural workers, with a view to providing insights quickly to UK policymakers regarding what support is required for this workforce as the pandemic continues. Within this remit, we also aimed to examine what specifically may have prompted particularly burdensome experiences throughout different pandemic stages, how these experiences have been perceived, and their consequences for mental health and wellbeing.

## Methods

This study was funded by Arts Council England (ACE) to be completed across a 6-month period, with the aim of sharing findings with ACE and the wider cultural sector via two reports throughout the research process to inform policymaking during the critical time of the pandemic (see Bradbury et al., 2022 for the final report). As traditional qualitative approaches would be too time-intensive, we employed a rapid qualitative approach, useful for research that “demands the sharing of findings in almost real time” (Vindrola-Padros et al., 2020). Such approaches have been used in response to major societal changes such as during previous epidemics (Abramowitz et al., 2015) and, more recently, in response to the COVID-19 pandemic to explore healthcare delivery (Vindrola-Padros et al., 2020). Specifically, we employed a longitudinal rapid appraisal qualitative approach, appropriate for examining processes of change and to “develop a preliminary, qualitative understanding of a situation” (Beebe, 1995). Whilst rapid appraisal is a relatively new methodology, it’s success in responding quickly to time-sensitive topics and collating actionable findings quickly to decision makers is acknowledged (Taylor et al., 2018; Vindrola-Padros et al., 2020).

The research involved two-stage semi-structured qualitative interviews with freelancers working in the cultural industries, with the first interviews taking place between July–November 2020 as part of wider research conducted for the UCL COVID-19 Social Study (https://www.covidsocialstudy.org/) and the second interviews taking place between May–July 2021 for this follow-up study. The first study explored participants’ lived experiences of the pandemic and any socioeconomic and psychosocial impacts (May et al., 2022), with the current study focusing specifically on perceived changes in experiences and circumstances over time. The interview guide was adapted for the follow-up interview to ask about changes to the working and social lives of participants since the first interview, such as the impact of different stages of the pandemic on working conditions and job opportunities, and the impact of these changes on mental health (see the topic guide in Appendix). The interviews lasted an average of 45 min and were conducted via either video or telephone by researchers with backgrounds in social science (KW, TM).

### Participants

20 self-employed freelancers working in the cultural sector from across the UK took part in initial interviews, with 16 of these freelancers participating in a follow-up interview. The remaining four participants were uncontactable following email invitations to participate. Convenience sampling was employed and augmented with purposive sampling to ensure diversity of professional role, gender, and age, with the study including people aged over 18 working within and across the performing arts, visual arts, and Film and TV industries. This approach was taken to respond quickly to the time-sensitive nature of the research, whilst also including individuals that would give us an understanding of how those working in different industries had been affected (see May et al., 2022, for further information about sampling). Participation in the research was voluntary, and all participants were provided with details either verbally or in writing about the nature and purpose of the research and what their participation would involve. Demographic details were also obtained via a short survey (see Table 1). Ethical approval was provided by University College London research ethics committee [Project ID 14895/005] and all participants provided written informed consent.

**Table 1.** Characteristics of freelancers included in the follow-up interviews for this study.

Number of participants	16
Profession	Contemporary Dance Teacher (1)
	Film and TV Producer (2)
	Independent Theatre Producer and Arts Administrator (1)
	Musician (4)
	Playwright/Theatre/Opera Director (3)
	Independent Production Associate – arts and film (1)
	Stage Actor (1)
	Screen Actor (1)
	Visual Artist (2)
Age	22–53 (35.4)
Gender	Male (5)
	Female (11)
Ethnicity	Black Other (1)
	Indian (1)
	White & Black British (1)
	White & Black Caribbean (2)
	White British (9)
	White Other (2)

### Analysis

As is typical of Rapid Appraisal, data collection and analysis took place concurrently (Beebe, 1995). This involved using RAP sheets (Rapid Assessment Procedure) after each follow-up interview to immediately document, and begin interpretation of, the interviews. The RAP sheet consisted of a table with thematic headings of interest based on the topic guide, along with a column for researcher notes, which was used to guide notetaking and systemisation of what was discussed. These RAP sheet summaries were then considered in regular team discussions (KW, TM, and AB), alongside listening to relevant sections of the interview audio, to explore and map emerging findings. Key themes from the RAP sheets were then charted into a new table by KW to explore overarching thematic patterns, which were discussed with the team (KW, TM, and AB) and used to guide the write-up of the key findings. In addition to the analysis of the RAP sheets, information that participants shared in their interviews about the financial support they had received and their changing employment status across the two interview timepoints were charted in an excel spreadsheet alongside demographic factors to search for patterns of change. Given how interviews were recorded and analysed in view of the rapid methodological procedure, the majority of the quotes included are paraphrased, rather than verbatim. However, some key quotes were transcribed verbatim and this is indicated throughout.

## Results and discussion

**Figure 1.**
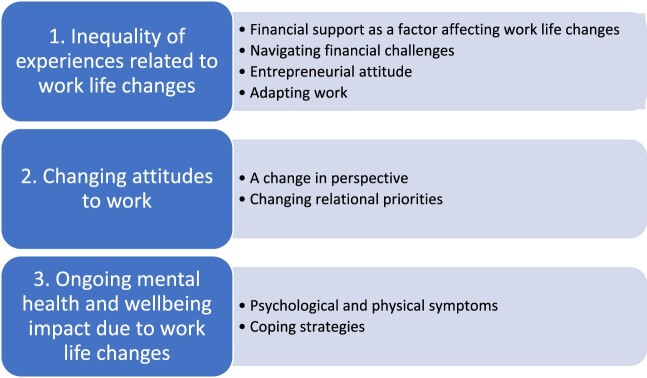
Themes and subthemes constructed from the rapid analytic process

Our data presented a picture of perceived changes to socioeconomic and psychosocial adversities for our sample of freelance cultural workers. We explore these changes and why they may have occurred through three themes and eight subthemes which were constructed through our analysis procedure, as presented in Figure 1.

### Theme 1: inequality of experiences related to work life changes

The extent to which participants’ working lives changed since their first interview varied, illustrating an *inequality of experiences* across our participants. Some participants reported only minor changes, stating that they were “still at a standstill” (ID1), and that work life had “not changed a great deal” (ID3), or “not changed much” (ID22) since their first interview. Others described fluctuating changes during this period, with cultural work opportunities coming back after being “non-existent last time” (ID5) and being like “feast and famine” (ID20). A selection of participants reported major changes to their working lives, for example obtaining full-time employment in the arts and moving away from freelance work (ID9, ID21):

Yes, I have now started working full-time, which I would never do, if it wasn’t for the pandemic. So, now, jumping from freelance, now I work 11 hours a day, five to six days a week … it’s given me more thoughts about how much I don’t want to work full-time, anywhere. (ID21, Film & TV Producer, quote transcribed verbatim)

Others reported that they left the cultural industries completely, such as setting up their own business [not directly related to the arts] (ID17), working in care (ID12) or teaching (ID11). For those who made these major changes, some reported considering staying in their new roles indefinitely (ID12, ID17), while others hoped to move back into an arts career, if they could secure arts project funding in the future (ID11).

#### Financial support as a factor affecting work life changes: “the government has prioritised office staff and buildings”

In this first subtheme, we explore how the inequality in changes to participant’s working lives could, in part, be explained by the amount of financial support acquired during the pandemic. Those who were ineligible for government support schemes due to narrow eligibility criteria were more likely to seek employment outside of the arts or to become unemployed.^2^ Of the five people in our study who decided to shift completely into non-arts work or were left unemployed, two never received government funding, one received funding but it was much later than needed and perceived as not enough due to mixed Pay As You Earn (PAYE) and freelance work, and two received no support at the time of their first interview but had managed to secure a small grant by interview two, by which time they had already sought alternative employment (see Table 2). Further, two of these participants had childcare responsibilities at home and one was an informal carer for their partner, making it difficult to secure work and leaving them in greater need of financial support. These participants perceived that the government had “prioritised office staff” (ID12) and were “a bit blind to [the] self-employed” (ID19), resulting in situations of financial uncertainty.

**Table 2.** Funding received by participants who left the cultural industries or became unemployed.

Participant ID	Gov funding interview 1	Gov funding interview 2	National arts council funding	Other funding	Changes to career reported at time of second interview
13	No	No	No	No	Part-time non-arts work
19	No	No	No	No	No arts work/unemployed
12	Yes	No	Yes	No	Part-time non-arts work
11	No	Yes	No	Local authority	Part-time non-arts work
17	No	Yes	Yes	No	Full-time non-arts work

Those who began the pandemic in an insecure position continued to experience increasing hardship as the pandemic continued, as has been acknowledged elsewhere (Banks & Connor, 2020). Participants who did not receive adequate funding initially or who were ineligible (and more likely to be already working within a landscape of uncertain job prospects) were those who either left their artistic career to work in a different sector or were left unemployed. Even when small amounts of funding or financial support were acquired later, these adversities were too great to sustain their previous freelance cultural careers.

#### Navigating financial challenges: “I took on temporary work for 3 months”

Several participants managed to balance small amounts of government support (e.g. Self-Employment Income Support Scheme (SEISS), and support from ACE and Creative Scotland) or other support (e.g. charitable funds) with cultural work to try and acquire the level of income they had pre-pandemic (ID22, ID10). However, for some, limited or no funding combined with reduced cultural work opportunities meant having to take on supplementary work from outside of the cultural sector. This included working in supermarkets, recruitment, and doing administration or charity work (“I took on temporary work for 3 months at [the supermarket] overnight stacking shelves”, ID9). Three participants also received universal credit (ID2, ID5, ID21), all of whom were aged 26 and under, suggesting that younger freelancers needed additional financial support, with many “fall[ing] through the gaps” of the SEISS eligibility criteria, as has been reported elsewhere (Comunian & England, 2020). After making the decision to take on a full-time job despite not wanting to do so, one of these participants (aged 24) noted, “I don’t ever want to be in a position where I have to apply for Universal Credit again”, feeling that they didn’t want to be “reliant on someone else” (ID13) for their financial support. Further, participants who were older in our sample had sources of additional support (e.g. long-term partners providing financial support); whereas all three of these younger freelancers were living in shared houses, with one having to move in with their parents to survive financially during lockdowns. The working environment in the cultural industries for young people was challenging prior to the pandemic, with many working for free and working long hours to secure work, as Hesmondhalgh and Baker (2011) have shown. However, our findings suggest that the pandemic exacerbated these challenges, with younger participants facing additional financial burdens and disruptions to their careers.

Several participants also noted that completing funding applications was akin to unpaid work; they required time and labour (“*there’s no time, they won’t fill in the forms for you*”, ID12), with little feedback provided when applications were unsuccessful. One of our participants who fitted in project applications around their childcare responsibilities explained:

I’ve been putting in the odd application for Arts Council funding. It’s just been six, eight months [of] rejections really on projects, and funding applications. So, yes, a fairly negative experience really … I was rejected … with not really any feedback, only that they preferred other applications. (ID19, Visual Artist, quote transcribed verbatim)

The national arts councils allocated funds for freelance cultural workers, but it was perceived that only those with the luxury of time to complete these applications were able to be in the running for receiving it. One of our participants who ultimately left the cultural industries during COVID-19 noted, “I cannot get into the Arts Council system” (ID12), explaining how she had to prioritise doing paid work and looking after her partner over filling in funding applications. Her language of “getting into” is also telling of how she perceived there to be an “in group” and an “out group” to Arts Council funding structures, viewing it as exclusory for those outside its existing networks, rather than an open fund for all. These beliefs about ACE and the value systems behind their funding structures had a real-world impact on her decision whether or not to remain in the industry.

Completing funding applications was also perceived as affecting income that could be attained elsewhere. One of our participants had previously secured a project grant from a non-departmental public body prior to the pandemic which was taken away (“lost the funding”, ID11) due to the challenges of carrying out the work during COVID-19, leaving them frustrated and seeking alternative employment outside of the sector. They reported putting in “so much planning” [for a project application] and “never being paid for any of it” (ID11), thereby affecting the income they could have gained from alternative work.

#### Entrepreneurial attitude: “A weird silver lining”

Cultural work has been characterised by an entrepreneurial ethos (Scharff, 2017). Drawing upon a Foucauldian approach, this ethos has been argued as extending beyond economic enterprise, whereby cultural workers become entrepreneurs of the self (Burchell, 2006; Foucault, 2008; Gill & Pratt, 2008; Scharff, 2017). On this view, entrepreneurialism may be argued as interconnected to neoliberalism, whereby individuals are compelled to become autonomous, demonstrating self-initiative and self-improvement (Foucault, 2008; Scharff, 2017). As Scharff states, “the neoliberal self is an entrepreneurial subject” (p.16) (Scharff, 2017). It could be argued that the majority of our participants demonstrated such an entrepreneurial attitude to their work in some way, as may be typical of the nature of a freelancer career which requires one to be autonomous and self-directed (Quader, 2021; Storey et al., 2016). However, what was particularly interesting in our analysis was that those who were Black, Indian, or from a mixed ethnic background created their own cultural opportunities (paid and unpaid), rather than relying solely on freelance contracts, employment (part or full-time), or supplementary work. The exception to this was one white female participant who created music opportunities as part of their new non-arts role in the care industry. Whilst entrepreneurial traits may create opportunities, they also exacerbate inequalities, whereby those who are marginalised engage in self-exploitative behaviours, increasing processes of individualisation and decreasing opportunities for solidarity.

Of the five people in our sample who were from an ethnic minority group (see Table 1), four self-initiated new opportunities, including setting up a series of online and doorstop concerts during lockdown (ID13), setting up a new freelancer networking scheme to build social capital (ID7), undertaking dramaturgy training to upskill and thereby attain freelance dramaturgical work (ID20), and setting up a new business (ID17). All of these participants were also female. In view of this, when looking across our whole sample and the different ways in which our participants adapted their work to survive during the pandemic, we also found that female participants tended to adapt the kind of work that they did to remain working in the arts (e.g. changing from theatre to film), while four of our five male participants described taking on supplementary work outside of the sector to survive (e.g. cleaning, working in a supermarket), made no change, or became unemployed.

Although our sample is small and we spoke to more women than men, our findings suggest that those who were Black, Indian, or from a mixed ethnic background and those who were female may have been able to adopt an entrepreneurial attitude to uncertainty, as has been explored by Scharff (2017) and White (2017) in the context of gender and racial inequalities respectively. Women have been portrayed in society as ideal entrepreneurial subjects even before COVID-19 (Gonick, 2006; Ringrose & Walkerdine, 2008; Scharff, 2017), and the pandemic has created conditions that have incited freelance workers to further cultivate their entrepreneurialism to survive increasing precarity (Szostak & Sułkowski, 2021). Further, in our first-stage interviews, we found that some participants maintained a positive attitude to the government’s pledge to provide further financial support for cultural workers, aligning with the problematic rhetoric of needing to be “resilient” in the face of economic uncertainty that shifts the responsibility of survival from governmental structures to individuals. By our second interviews, this hope in financial support had waned; however, the ability to demonstrate resilience accelerated, with these participants becoming “paradigms of entrepreneurial selfhood” (Ross, 2008), whereby they were able to quickly adapt their cultural work to the new social conditions of the pandemic (“I’m like … I’m an entrepreneur! … I definitely want to build the business”, ID17).

In view of the racial and gender inequalities previously documented in the cultural industries (Brook et al., 2018; Brook et al., 2020; Scharff, 2017), and evidence showing that existing inequalities are being exacerbated by the pandemic (Cultivator, 2020; Spiro et al., 2021), it seems possible that individuals who are consistently marginalised in the sector and had learned the skills to demonstrate self-initiative to attain work in the sector pre-pandemic (White, 2017), were also readily able to pivot their practice, and act resourcefully and entrepreneurially when the pandemic hit (even if this meant self-exploitation via unpaid opportunities). Of note, one of our female participants who was from an ethnic minority group set up their own full-time business with colleagues from outside of the sector and reported this as “a weird silver lining” of the pandemic (ID17). And a female participant who acted as “kind of a carer” (ID12) for their partner living with disabilities who moved into a non-arts job remarked that their “accidental career is more rewarding than their deliberate [arts] one”. These two participants adopted a grateful attitude to their unexpected changes, drawing upon self-initiative and resilience as their go-to behavioural responses to precarity and structural inequalities within a neoliberal culture.

#### Adapting work: “Art is the opposite [of] preparing for a zoom meeting”

Connected to an entrepreneurial attitude, many participants in our sample who stayed working in the cultural industries had to change the type of work they engaged in, relating to reduced options of cultural work since their first interview and the need to adapt face-to-face or performance work to pandemic restrictions. For example, moving from music and modelling into film as this industry came back after the first lockdown sooner (ID5), a greater emphasis on opera directing, rather than theatre, due to longer preparation periods without performances (ID7), stage acting into screen work and dramaturgy (ID20), and focusing more on dance teaching, rather than dance performance (ID10). One participant expressed letting their work as an artist “drop off” to focus more on publishing because “that’s where the money is” (ID3). This was similar to another participant who decided to focus on more “business orientated” work (e.g. business plans, strategic planning) in place of face-to-face cultural work (ID4). A composer also expressed that there was “less choice in the work overall” with more opportunities occurring in TV than in other industries (ID22), similar to another performer who previously worked across stage and screen who shifted into TV and film (ID5). In our initial research, whilst experiences of adversities did vary, the issues described were primarily in relation to having no in-person work and the challenges of online working. Our study shows how these circumstances changed for people working in certain industries, supporting wider research noting that Film, TV, and publishing sectors are showing signs of “recovery and growth”, with other industries, particularly the performing arts, still struggling (Walmsley et al., 2021).

Interestingly, however, two participants did not want to attribute the blame for the changes to their freelance careers to the pandemic. One participant noted that they were experiencing “peaks and troughs of work” but that they were “not sure if it’s the pandemic” because their choice of industry often meant inconsistent flows of work (ID 22). Another participant who had recently had a baby reported increased financial challenges but indicated that she wasn’t sure if this was due to maternity leave, rather than the pandemic (“there’s other factors”) (ID 4). Whilst there could be other factors coming into play that disrupted work opportunities, with the cultural industries notorious for making it difficult to secure work and have a family (Scharff, 2017; Skillset, 2010), it also shows how these two participants resisted attributing the adverse changes they experienced to a fragile sector in a time of crisis. Instead, they chose to focus on their own lifestyles and choices as factors affecting their workflow. Aligning with wider research, they tended to feel that their career success was dependent on individual, rather than structural, factors (Brook et al., 2018), accepting responsibility themselves (Banks, 2007; Scharff, 2017).

Another dimension of adapting work was in relation to lifestyle. In their first interview, the majority of participants described working from home, with many using online methods to create work. At follow-up, many had adopted hybrid approaches, working both in-person and online, with in-person work requiring adherence to COVID-19 social distancing guidelines which made work “very strange” because “so much of the relationship is withheld” (ID20). One participant (ID2) expressed how the film & TV industry were particularly cautious:

I honestly say the film and TV industry was probably one of the most heavily, in a way, regulated with regards to protocols and guidelines, in regards to some other industries … So, we’d be working with masks all day, every day, you have temperature checks every day. Usually you’re doing an at-home test before you come into work. (ID2, Film & TV Producer, quote transcribed verbatim)

A number of participants also expressed growing tired of online working and trying to move away from it completely, reporting zoom fatigue (ID22, ID17, ID7, ID4) and noting the limitations of doing cultural work online because “developing art is the opposite to preparing for a zoom meeting” (ID7). Whilst these challenges were mentioned in the first interviews, they were reported to have intensified in the follow-ups with participants describing an increase in frustration, even in the context of work that was more suited to online platforms (e.g. publishing, teaching). This raises questions about whether “going online” is a viable option for certain industries where in-person aspects have traditionally been focal, as has been highlighted in wider research. For example, Spiro and colleagues (Spiro et al., 2021) found that, for those working in the performing arts during the first UK lockdown, adapting work online was not always possible and was a “poor substitute for face-to-face activities”. Research on the impact of the pandemic on arts residencies also shows a similar pattern to our findings (Res Artis and UCL, 2020, 2021).

Thus, whilst a freelance career may seem like an occupation that will always entail uncertainty with regards to job security, the extent of this insecurity varies considerably, and funding structures could have considered this further.^3^ There are different dimensions to precarity as has been argued by Rodgers and Rodgers (1989), and one of these dimensions during COVID-19 has been the industry worked within and the ability to adapt work during different stages of the pandemic. During times of “opening up” after lockdowns, for example, precarity varied dramatically for freelancers because only certain people were able to transition back into in-person work (e.g. those working in Film & TV).

### Theme 2: changing attitudes to work

As we explore in this second overarching theme, a number of our participants also expressed changing attitudes to work as a result of the pandemic, connected to the de-globalisation of professional relations and an increase in working from home and time spent with family.

#### A change in perspective: “I now place more of a focus on family and friendship”

Participants reported adopting a “different perspective” (ID3), where “time to think … fundamentally changed priorities” (ID9). One participant noted that this meant they had a new sense of purpose and a realisation that they preferred their new career working with colleagues from outside of the cultural industries:

… having this new kind of sense of purpose in what I’m doing. One of my friends recently […] said “you look younger” “cause like a weight’s been lifted – suddenly I’m like, ah this is so much more fun”. (ID17, Independent Theatre Producer and Arts Administrator, quote transcribed verbatim)

Whereas previously the focus of life was primarily on attaining and sustaining a career in the arts, focus shifted to home, family, friendships, and pets (ID9, ID11), with participants also reporting preferring a “slower pace” to life that is “less hectic” (ID3, ID11) where “simple things” are viewed as “important” (ID22):

I’ve changed priorities and learned to live frugally. I don’t care if something is tatty … I want to focus on what’s important – home, family, pets, friends … just need enough money to choose life we want to live and be happy and healthy … I don’t want to compromise happiness. (ID11, Independent Production Associate - arts and film)

One participant (ID9) also described going through a “trial period of living with [their] partner” during the pandemic; the participant stated that he wanted to “contribute equally and put down foundations” in relation to house ownership, sharing that his focus had shifted from a freelance career in the arts to attaining a full-time job which would provide the financial stability for these new aspirations with their partner:

I now place more of a focus on family and friendship, whereas before it was creativity and the arts. (ID9, Playwright/Theatre/Opera Director)

Shifts in perspectives were also connected to reduced motivation to commit to working in the arts (ID9, ID19) – which was perceived as a precarious industry to continue working within – resulting in “lost drive” and “less ambition” (ID3).

These attitudes are markedly different from what has been theorised and documented prior to the pandemic. Scholars such as Ursell (2000) and Kuehn and Corrigan (2013) have argued that cultural and creative workers engage in “hope labour” whereby they may work for free or make other sacrifices such as working very long hours in the hope that this labour will result in future work opportunities and career success. Although we saw elements of this behaviour in our sample, with some engaging in unpaid work, by the second interviews this was being questioned and attitudes and priorities were shifting. Duffy (2016) and Gerber (2017) have also suggested that such self-exploitative actions may be engaged in as a trade-off for creative autonomy, whereby flexible working is valued more than income. Thus, the “freedom” promised by the sector and the hope of future reward has been a key motivator for cultural workers in the past, but the pandemic seems to have disrupted this value system, with workers placing greater emphasis on non-work time and personal relations during the pandemic.

Thus, for some, the pandemic seemed to unveil the inherent instability of the sector and lack of job security that it offers, prompting workers to reassess their life goals and where they derive meaning. These findings align with Durkheim’s theorisation that an unexpected change in circumstances (e.g. change to employment or financial losses), may result in one questioning their moral beliefs and values because “he sees nothing above him to which he belongs”, whereby the stability of a shared collective consciousness is disrupted (Durkheim, 2002/1897). In this case, moving away from the shared value system of the cultural industries (i.e. the collective consciousness) and constructing values in different relational settings (i.e. with non-arts colleagues or as part of home life). Relations to others can therefore be viewed as deeply interconnected with changing attitudes.

#### Changing relational priorities: “engaging more with family”

Where pre-pandemic social capital was important within cultural networks to secure work and attain symbolic capital to achieve career ambitions (Gerber, 2017), a selection of our participants displayed an emphasis on creating or building on personal relations for fulfilment during COVID-19.

The majority of participants reported the connections with people with whom they lived and those who lived locally becoming stronger. Changes in local connections were also described, with one participant using online networking to make new friendships in her local community. This translated into in-person relations, creating a “change for the better”, stressing a feeling of “being more embedded in the community” (ID3). Another participant noted feeling “happy in their neighbourhood network” (ID21), but also “a bit exploited” by neighbours who they had helped out during lockdowns (ID21). Many also reported feeling closer to their romantic partners (ID19, ID1, ID22, ID9, ID21) who were identified as a key source of social support. For example:

I didn’t really need other people, because I really enjoyed the relationship with my boyfriend and the company, the partnership that we’ve shared … So, I wasn’t lonely in the pandemic, and I think in comparison to many other freelancers, who are single, it made a massive difference that I had all my social needs covered. Because I lived with my favourite human and my best friend, who is also my boyfriend, so, I think, in terms of social needs, I had all of them covered. (ID21, Film & TV Producer, quote transcribed verbatim)

In terms of professional networks, participants reported the loss of their professional networks in their first interview due to being unable to meet colleagues in-person during the first UK lockdown, with many of these colleagues also considered as friends. For those who experienced a changing attitude to work, this loss was sustained and, for some, intensified. This resulted in a strengthening of their personal relations, while work colleagues and those who did not live locally became more peripheral in their network. Participants reported “engaging more with family” (ID1, ID3), and seeing work colleagues less (ID22, ID1, ID19, ID11, ID21), resulting in “smaller social circles” (ID9), “feeling cut off” (ID11), and limited socialising (ID21, ID7).

Other participants increased their professional social network through networking online (ID13) and reported strengthened colleague relations due to a sense of shared experience of pandemic-related challenges and “time to talk” (ID20). In addition, the opportunity to meet in person when restrictions eased was viewed as a way to “socialise” (ID5) “come together” and “cement” work connections (ID10), with honesty, vulnerability and shared experience viewed as facilitative of this coming together (ID7, ID13, ID21). However, these shifts were described as complex, as the pandemic “strengthened the friendship aspect of some arts connections and weakened others” (ID3), with work relations viewed as “up and down” (ID5), suggesting professional networks were unstable. Further, the sense of connection to arts organisations was considered to have weakened, with participants feeling let down by organisations (“*feeling sort of thrown out”,* ID1) due to “lack of contact” and a feeling of “organisations versus freelancers” (ID7). Namely, there was a sense of a “divide in the community” where arts organisations were “not considering freelancers” (ID7). For some, social media exacerbated this, with online networks viewed as “divisive” and “fragmented”, with organisations promoting a message “that everyone is back at work in the arts” (ID9), when “it’s only half the workforce” (ID12).

In sum of this theme, a number of our participants shifted their priorities as a result of the pandemic, changing their focus from professional development to personal life. This was interconnected with changing relations, with professional networks viewed as unstable and many increasing their time with family and friends.

### Theme 3: ongoing mental health and wellbeing impact due to work life changes

For a number of our participants, ongoing work and financial challenges alongside changing attitudes led to exacerbated mental health symptoms, as we explore in this third theme. Nonetheless, others derived enjoyment from their “new normal”, aligning with their shifting priorities and changing attitudes.

#### Ongoing psychological and physical symptoms: “it doesn’t matter if I just go to bed”

Several participants reported an increase in stress and anxiety in their first interview, and this was found to be further exacerbated during subsequent lockdowns, often manifesting as *psychological* symptoms. This included an increase in anxiety (ID20, ID22, ID12), panic (ID1), stress (ID12, ID11, ID21, ID5), depression (ID20), rumination (ID20), a feeling of the mind racing (ID9), claustrophobia (ID10), reduced self-esteem and confidence (ID12, ID9), and reduced overall wellbeing and quality of life (ID20, ID3, ID9, ID11). One participant also reported feeling suicidal “last year” (in 2020) but, having since secured a job outside of the cultural industries, described feeling more positive about the future (ID12). Many also reported ongoing existential questioning relating to their sense of identity, progression, and purpose (ID12, ID1, ID20, ID2). For some, this resulted in feeling hopeless (ID1, ID7), out of control (ID12, ID1), and finding it hard to discern time during periods of no work (ID7). The lack of work was also perceived as “heart-breaking” (ID11, ID21), and left one feeling that “it doesn’t matter if I just go to bed” (ID20), with another expressing that the lack of funding received for work was “demoralising” (ID19). These psychological experiences also connected to a range of moods, including feeling guilty (ID1), low (ID2, ID3), worried (ID9, ID13, ID21), devastated and bereaved (ID7), shameful (ID9), sad (ID20, ID9), paralysed (ID20), lost (ID10), depleted (ID11), and, in relation to the lack of support from government, betrayed (ID11). Many also experienced increased anxiety or worries relating to the uncertainty of the future (ID3, ID7) and were unable to see any “prospects” or “positives” to the changes to their career (ID20). Despite many worrying about the future of their professional lives in the cultural sector, a number of participants, however, said that they would continue to pursue their career in the cultural industries (ID1, ID5, ID9, ID13, ID12). Several also connected this to their identities, stating that “staying in the arts is not a choice” (ID11) because it’s “integral” (ID1) to who they are.

For several participants who were able to secure work opportunities (either from outside the sector or in-person work in certain industries during times of opening up), their negative affect was also experienced alongside more positive feelings, with the pandemic period described as a “rollercoaster of emotions” (ID3). This meant that negative feelings “came in waves” (ID9), with feelings such as being “excited” about the future (ID9), “positive” since work returned (ID2), and feeling “thrilled” about future projects (ID7) also part of this “turbulent” time (ID3). Moreover, these positive feelings increased for some as work opportunities returned, with one individual reporting feeling “better over time” (ID5) and suggesting that they had become “more resilient” through the pandemic (ID11) and had “not given up the joyful, creative part of myself” (ID21). One participant who had left the cultural industries to start their own business also reported enjoyment from doing so:

Yeah well I’ve started my own business since I last spoke to you … And just really love it, really enjoyed it found it really rewarding, and it just confirmed that I wanted to do more work around that. (ID17, Independent Theatre Producer and Arts Administrator, quote transcribed verbatim)

The negative aspects of this instability, however, had a dramatic effect on some, also displaying as *physical* symptoms. Whilst in their first interview physical symptoms were reported (e.g. sleep disturbances), these were exacerbated, with new symptoms occurring. Two participants described ongoing stomach pains (ID5, ID9) due to the “impact of work changes” (ID5), with one (ID9) describing these as “IBS bouts” that they didn’t have before the pandemic (“not a problem in the past”, ID9). Another said that their physical symptoms had continued to increase since their first interview and included “heart thumping, sweaty attacks” and “a mild electric shock feeling” that also “affected memory really badly” (ID12). This was a result of what she described as the “stress of hanging onto [their] old career” (ID12) as they were transitioning into a new job outside of the sector. The increased usage of Zoom for work also led to a new “physical reaction” for one participant, making her feel sick (ID7). In relation to sleep, several reported not sleeping well (ID10, ID12, ID9), or feeling lethargic (ID7, ID9, ID12), but one participant said their sleep had improved since their first interview. This participant had moved to a new house to remove “environmental stressors” (ID22).

The health impact described was perceived by participants as connected to work life changes. However, in view of the passion that participants described having for their pre-pandemic cultural work (“music my passion” ID5; “it’s a hobby, passion, and a profession … part of me” ID1), it’s possible that these impacts could also be connected to changing attitudes and increased “existential questioning” of professional identity (ID1, ID22). There is a wealth of literature published pre-pandemic showing that many pursue a cultural career (particularly artists) because it is akin to a vocational calling (Gerber, 2017), relating to it being satisfying or pleasurable (Bain, 2005). Being an artist is viewed as providing meaning and purpose (Lingo & Tepper, 2013), even considered a “sacred” profession (Simpson, 1981) providing “a necessary ritual for artistic self-actualization” (Hooks, 1995, p. 128; as cited in Bain, 2005). Looking to the wider literature on career transitions, changes to identity as a result of these transitions have been viewed as both positive (growth experiences) and negative, such as in relation to detrimental health repercussions (Oakland et al., 2013). The picture presented in this study, however, is not clear. On the one hand, we found a range of adverse mental health consequences with some unable to see their career shifts as “positive”. But, on the other hand, many reported finding meaning in new ways, with their relationships with certain people (such as romantic partners) flourishing. We cannot make a normative assumption about a career in the cultural industries for this group as the only source of wellbeing, nor suggest all participants “should” try to get their pre-pandemic work lifestyles back as we return to a state of “normality” (i.e. as the UK transitions to cultural production sites operating without any restrictions). Indeed, for some, their newfound directions seemed to provide a subjective fulfilment that they did not have before.

In fact, those unable to renegotiate their identities may be putting themselves in a more vulnerable position in the longer-term because viewing their cultural careers as “not a choice” could mean continuing to pursue their career in turbulent conditions, even if it could have negative psychological consequences. As explored by Gross and Musgrave (2020) in the context of professional musical identities, the “non-musical” identity is “frightening” and associated with non-existence, with musicians preferring to continue to develop their musical identities over having to construct a “non-musical” one, despite the associated adverse mental health consequences of this decision (pp. 53-54). Further, in the context of the pandemic, those people whose working lives are not yet “back to normal” may also suffer mental health distress from the idealised image that the future will mean “building back better” as per government rhetoric. Some cultural organisations publicly sharing their success stories of “going back” online, such as via social media, may present an image of growth and positivity as the norm, when a large proportion of freelancers, and the sector at large, are still acutely experiencing the pandemic’s impact. This combination of the individual’s commitment to achieving a cultural career despite adversities and the presentation of opportunities available builds on Menger’s (2014) theory of art and achievement under uncertainty. Whilst uncertainty can fuel innovation, it can also present a delusion because the prospect of achievement in the cultural industries is enough to motivate individuals to continue to pursue their careers, even if there are very few opportunities and the likelihood of “making it” is minimal. Thus, the construction of “post-lockdown” success shared by major arts and cultural organisations may provide hope for the individual freelancer that their pursuits are worthwhile, when opportunities may be limited, resulting in detrimental health impacts in the longer-term.

#### Coping strategies: “only so much yoga and baking you can do”

In order to manage poor mental health, a number of participants reported changes to their *coping* strategies since their first interview. Several described feeling that they had reached a peak of recreational activities and were no longer experiencing these activities as protective for their mental health. One participant shared that there is “only so much yoga and baking you can do” (ID10), with another saying that the “novelty” of ordering food and subscriptions online “ran out” (ID21), and others reducing activities such as knitting and sewing (went into a “*knitting frenzy”; “half done sewing projects”; “slowed up”,* ID20), mindfulness apps (“*using headspace less now”,* ID9), exercise (ID13), and yoga (ID3). Thus, the kinds of self-help strategies that participants tended to reduce were those that were consumption-based and potentially costly such as mindfulness apps, ordering takeaways, and yoga. As has been argued by Davies (2016), when wellbeing is embedded in transactional business logics, it falsely sells wellbeing as something that can be transacted or purchased, particularly at an individual level. A number of participants also said that they had actively reduced their intake of social media, the news and TV to support their mental health (ID5, ID9, ID3, ID12), aligning with wider research showing that following COVID-19 news or doing other screen-based activities is detrimental to mental health (Bu et al., 2021b).

Instead, coping strategies that involved self-reflection or engaging with other people and nature were perceived as more helpful, aligning with participants' changing priorities. A number of participants described continuing to use coping strategies such as sea swimming (ID1) and walking (ID22, ID17, ID3, ID9). Participants also used new self-help strategies such as engaging in developing their self-awareness (ID17), goal setting (ID20), and diary writing:

I’ve been keeping a diary, and I stopped doing that a few weeks ago. But from the beginning of January up until maybe halfway through April, I was writing a diary every day. Not a big long thing of what I’d done the day, but just bullet points, just to write it down on a page. Because I was saying last time, I think, to [other researcher], as well, I was just losing track of time. There were times I just didn’t know what day it was. (ID1, Musician, quote transcribed verbatim)

Several also drew upon formal support. One participant started medication for their mental health when they “realised the pandemic would go on” which supported them in having “energy to fill in job applications for other work” (ID12). Others described continuing with therapy sessions which were “amazing support” (ID3), or finishing therapy but continuing to use “the tools learned through it” (ID22).

In sum of this theme, a number of our participants experienced increased psychological and physical symptoms as a result of their changing working lives and the consequences of these changes on their professional identities. Some participants were able to find fulfilment from career changes, but others continued to pursue their cultural careers despite limited opportunities and mental health challenges. Participants found that consumption-based coping which had been helpful at the start of the pandemic was no longer supportive, with relational and nature-based activities more fruitful to supporting mental health.

## Implications and conclusions

This study aimed to explore how perceived socioeconomic and psychosocial experiences have changed or evolved for freelancers working in the cultural industries since the start of the COVID-19 pandemic in the UK. It shows how ongoing adversities manifested as changes to work life, with some experiencing small changes (e.g. to the kind of work carried out) and others experiencing major changes (e.g. leaving the sector to pursue alternative employment), as well as varied changes to social lives. The amount and type of change experienced varied depending on eligibility and amount of government financial support received, industry worked within and the opportunities available to adapt working practices, caring responsibilities, and demographic factors such as age which also intersected with career stage and lifestyle choices. Although a small sample, there were also indications in our analyses that those who were Black, Indian, or from a mixed ethnic background and those who were female felt that they needed to adopt an entrepreneurial attitude to uncertainty, thereby leading to the reinforcement of neoliberalism and exacerbating inequalities. For some participants who experienced major changes in relation to the kind of work they pursued or to their working environments, changing attitudes to work were seen. This manifested as fewer “careerist” values in relation to their commitment to working in the cultural industries and greater emphasis on personal relations, such as family life. As a result of these major changes to work and social life which were also linked in with our participants’ professional identities, many experienced ongoing or increased negative psychological and physical symptoms.

Our research brings into sharp focus the need to question what “cultural value” is in the context of the pandemic (and how it may differ from pre-pandemic times), and to further explore the valuing processes of freelance cultural workers. Although pre-pandemic there has been much discussion about the need to move away from an economic understanding of cultural value and an “absolute cultural value” that could be “captured” in favour of a more processual conception of valuing processes (Belfiore, 2015; Stevenson, 2013; Walmsley, 2016), there has been little discussion of how the valuing processes of those working in the cultural industries may intersect with wider life experiences and attitudes. Our study highlights that the pandemic can enable the construction of different priorities for some freelancers, whereby a cultural career needs to coexist, and be compatible with, other shared valuing processes, such as those engaged in with family, friends, or partners. This has a major implication for cultural policy as it suggests that policymakers need to consider how to provide opportunities for cultural freelancers to express and live-out their new and shifting priorities, allowing freelancers to balance their career goals alongside their personal goals (which may also overlap). In the current political and sociocultural landscape of the UK, this is problematic as the financial support available for freelance cultural workers is inconsistent (i.e. an emphasis on one-off project grants), with the sector also typified by inequalities in the workforce (Brook et al., 2018). Freelancers undertake precarious cultural careers that make it difficult to attain the kind of personal goals that our participants described which would require stable income (e.g. owning a house, having a family, looking after pets), with additional barriers for certain groups due to structural inequalities (e.g. demographic factors). If policymakers wish to ensure fair and equal opportunities, they need to consider both the changing valuing processes of freelance workers and the financial support required for this workforce to maintain their careers in view of changing attitudes to work – the “new normal”.

Interconnected to this, our research supports wider research showing that the pandemic may have exacerbated existing inequalities in the sector (Cultivator, 2020; Spiro et al., 2021), particularly in relation to gender, ethnicity, age, and caring responsibilities. Whilst a limitation of our study is that our convenience sampling approach meant a dominance of female, white participants and we did not collect numerical data relating to income or years worked in the sector, the inequality of experience that we found embedded within our sample suggests a widening of financial inequality for freelancer cultural workers which also led to adverse mental health impacts. It is vital that policymakers consider how to provide extra financial and psychological support for those struggling to survive in the sector, considering not only loss of income due to the pandemic (i.e. as per the Creative Scotland Hardship Fund) but how changes to lifestyle may impact upon mental health, the ability to undertake work (e.g. for those with caring responsibilities at home) or fill in funding application forms (e.g. for those undertaking non-cultural work to survive). Funding criteria needs to be more nuanced and open to a range of complex situations that have occurred as a result of the pandemic, with funders also considering how to ensure their funds are accessible and perceived as open to all.

Another implication is in relation to the future of online working for freelance cultural workers. In view of the challenges of online working, a range of reports seeking to inform organisational strategy or policymaking in the future have suggested that resources are needed to support with how to move cultural events and workshops online (Culture Counts, 2020), provide opportunities for freelancers to upskill including digital training (ScreenSkills, 2020), and conduct research to identify and remove “barriers to entry into the digital market” (Department for Digital Culture Media & Sport, Arts and Humanities Research Council, 2021). However, this study suggests that we not only need to answer the question of how to support this workforce to “go digital” but also whether we should be doing so. If there are certain kinds of cultural production (notably, performing arts production) that are not so well suited to online delivery, then future focus should also be on alternative ways of working that are innovative and economically viable at times when traditional ways of engaging (such as in-person at concert halls, theatres, and stadiums) cannot happen. This is a challenge, but there has been a selection of audio-based, socially distanced, and “blended” projects since the pandemic began, which suggests that there may be innovative options to develop and broaden the concept of “the digital” in the future (Child et al., 2021). Viewing the rapid increase of mental health impact explored in this study alongside the decline of autonomy in relation to kinds of work and expressed changing attitudes in relation to digital working, finding new offline or “blended” ways of working may be vital to ensure a motivated and healthy freelance workforce in the future.

The findings of this study are rooted in a foundation of diverse experiences, whereby the adversities of the pandemic affected freelancers in the cultural industries in a range of ways. Future support provided for this group therefore needs to be highly bespoke.

## Notes

1 We draw on the United Kingdom government’s Department for Digital, Culture, Media and Sport’s definition of the cultural sector as “those industries with a cultural object at the centre of the industry”; examples include performing arts, visual arts, museums, and galleries, and film, television, and radio (DCMS, 2021). We purposely do not use the language of the “creative” industries which is broader and has tended to be tied to celebratory or “positive” narratives about what these industries contribute to society, particularly in relation to digital technologies (Scharff, 2017; O'Connor, 2010).

2 The United Kingdom COVID-19 self-employment income support scheme required a record of tax returns and for freelance work to make up at least 50% of overall income for those based in England. This was perceived as narrow for workers in the cultural industries because newly self-employed individuals were ineligible and because many workers undertake mixed PAYE and freelance contracts (Comunian & England, 2020).

3 Whilst it was recognised in Scotland that freelancers needed extra financial support (e.g., Creative Scotland Hardship Funding), specific funds for freelancers have been limited elsewhere in the UK.

## Appendix

### Participant topic guide

#### Ask to describe “normal life”

- Can you tell me a little bit about your job and what it is you do, just as a refresher as it’s been a little while since your first interview?
- Type of work, hours etc,
- Full -time parent or carer?
- Who you normally live with, does this change, separated/ extended family?

### Work life

#### Can you describe how your working life has changed since we last spoke? (Remind them date of last interview)

- Describe a typical day now
- Describe your work environment now
- How much autonomy do you have in your role and how has this changed?
- Do you find your job rewarding?
- Do you feel able to do your job to a high standard? Has this changed throughout different stages of the pandemic?
- Do you enjoy your job?
- How able are you to follow organisational rules and how do you feel about this?
- Do you feel safe at work? In what way?
- How have your work practices changed? Have you adapted your work in response to Covid-19? Have you engaged in any online/digital work?
- Have you undertaken any extra work to supplement any income affected by Covid-19?
- Have you been or are you eligible for any government support/schemes in response to Covid-19? (evidence of PAYE, average trading profit, “adversely affected” by Covid, plans to continue operating into 2021 etc.)
- What impact have any changes to your work life had on you?
- [If any negative changes are expressed] What, if anything, has exacerbated these changes? (prompt: structure of the industry, precarity of arts careers, lack of financial support)

### Social life

#### How would you describe the relationship between your working life and your social life?

- Do you connect socially with anyone who you work with? (prompt: have you previously or recently found work through social connections? Has this changed during the pandemic? If so, how?)
- Have these relationships/ networks changed throughout the pandemic? If so, how?
- Has how you interact changed during different stages of the pandemic – face to face, online or social media?
- Do you think the pandemic has had an impact on the arts/cultural community? If so, how? How would you describe your role within this community? Has this role changed during the pandemic? If so, how?
- Describe your sense, if any, of community or disunity to the arts/cultural community at large during this crisis?
- Could you describe the social support you have? (such as emotional support, advice and information, someone to help you with practical essentials/to talk to about challenges in the arts/cultural sector) Has this changed throughout different stages of the pandemic**?** If so, what has been the impact of Covid-19 on your social support?

### Mental health

- How do you feel about the changes to your working life that have been brought about by Covid-19?
- Have they had any impact on your mental health or wellbeing? Please tell us about these.
- What are the things most bothering you at the moment (work or outside of work)?
- What have been the major triggers/causes of any mental health or wellbeing issues? (i.e. closure of arts venues, lack of financial or emotional support)
- How has [the provision/ lack of] government financial support/schemes impacted your mental health or wellbeing?
- Has the pandemic had any impact on how you derive meaning from your practice? If so, how?
- Have you experienced any impact on positive emotions? (prompts: how deeply you can engage with what you are doing, sense of meaning/ purpose, relationships with others, how well you are managing and feelings of control over your situation?)
- Has there been any impact on your sense of identity? If yes, how does this relate to your work identity?
- Have you experienced any negative psychological feelings? (prompts: such as shame, guilt, lack of pleasure, anxiety, worry)
- Please tell us about any physical symptoms due to being stressed or anxious? (prompts: fatigue, sleep problems, pain, illness symptoms, palpitations)

#### Have you been doing/ planning anything to help with this?

- How has your support been, from friends/family? From work colleagues/your organisation?
- Connecting with family or friends online
- Online groups?
- Hobbies/Reading/engagement with the arts
- Exercise at home <ask about what they have been doing and if there are specific resources they have found useful to exercise>
- Volunteering
- Other engagement

#### Why are you doing/ not doing these things?

- Helpful/ not helpful – please tell us why
- Enjoyable
- Good for mental health/wellbeing
- Can’t get online, not connected, not comfortable, affordability, confidence in using/ skills
- Skills in using the internet/communication software
- Living arrangements/Work/caring demands
- Peer support/ pressure
- Difficulties/ restriction in physical environment

### Prospection

#### What do you think are the implications of the pandemic for:

1. Your future in the arts/cultural sector?
  - Do you think there will be any changes to the way you work in the future? Why/why not?
  - Will you stay working in the arts?
  - Will the way you connect with others in your industry change or stay the same?
2. Has this changed any of your priorities for the future? **the sector at large?**
  - What are the challenges/opportunities for those working in the arts?
  - How might the pandemic impact upon audiences?

#### Has the pandemic meant that you have any worries for the future? Have these worries changed throughout different stages of the pandemic? If so, how?

- Worries about work/the future of your work?
- Worries for yourself? Anything not directly connected to work?

#### How are these different from the worries you had before?

- Sense of control/ powerlessness
- Severity of worries / perspective

#### Will this change the way you live your life in future?

- The way you connect with others
- How you look after yourself
- How you support others
